# Management of Medium and Long Term Complications Following Prostate Cancer Treatment Resulting in Urinary Diversion – A Narrative Review

**DOI:** 10.3389/fsurg.2021.688394

**Published:** 2021-08-09

**Authors:** Benedikt Hoeh, Stefan C. Müller, Luis A. Kluth, Mike Wenzel

**Affiliations:** ^1^Department of Urology, University Hospital Frankfurt, Goethe University Frankfurt am Main, Frankfurt am Main, Germany; ^2^Cancer Prognostics and Health Outcomes Unit, Division of Urology, University of Montréal Health Center, Montréal, QC, Canada

**Keywords:** prostate cancer, urinary diversion, radical prostatecomy, radiation theraphy, devastated bladder outlet

## Abstract

The purpose of this narrative review is to discuss and highlight recently published studies regarding the surgical management of patients suffering from prostate cancer treatment complications. Focus will be put on the recalcitrant and more complex cases which might lead to urinary diversion as a definite, last resort treatment. It is in the nature of every treatment, that complications will occur and be bothersome for both patients and physicians. A small percentage of patients following prostate cancer treatment (radical prostatectomy, radiation therapy, or other focal therapies) will suffer side effects and thus, will experience a loss of quality of life. These side effects can persist for months and even years. Often, conservative management strategies fail resulting in recalcitrant recurrences. Prostate cancer patients with “end-stage bladder,” “devastated outlet,” or a history of multiple failed interventions, are fortunately rare, but can be highly challenging for both patients and Urologists. In a state of multiple previous surgical procedures and an immense psychological strain for the patient, urinary diversion can offer a definite, last resort surgical solution for this small group of patients. Ideally, they should be transferred to centers with experience in this field and a careful patient selection is needed. As these cases are highly complex, a multidisciplinary approach is often necessary in order to guarantee an improvement of quality of life.

## Introduction

Prostate cancer is the most commonly diagnosed cancer in men, with an estimated 1.3 million diagnoses worldwide in 2018, ranking as the fifth leading cause of cancer death in men (1). Radical prostatectomy and radiation therapy can be seen as equally accepted therapeutic approaches regarding oncological outcomes and play a crucial part in the curative active treatment strategies for prostate cancer (2). In the last decades, less invasive surgical approaches, as well as focal therapy concepts, e.g., high-intensity focused ultrasound (HIFU), brachytherapy and cryotherapy became frequently discussed treatment strategies of localized prostate cancer due to a trend to minimize morbidity while providing maximum of oncological tumor control (3, 4). Moreover, multimodal therapy concepts such as combination of surgical/radiation approaches, salvage or cytoreductive treatments have shown improvement of the survival outcomes in settings of high-risk, locally advanced or even metastatic prostate cancer patients (2).

Regardless of the constant urge to improve treatment and minimize therapy-associated side effects, concomitant and late onset complications have to be carefully taken into account, when treatment decisions are made and should be carefully monitored and managed. Severity and time of appearance of persisting side effects differ regarding the underlying treatment and can result in a bothersome reduced quality of life for the patient (5).

The vast majority of complications following prostate cancer treatments across all stages can be successfully treated conservatively with a significant increase of patients' quality of life. Unfortunately, a small proportion of patients suffers of ongoing (chronic) complications, leaving patients, and Urologists in a bothersome and frustrating situation. Urinary diversion can be seen as an *ultima ratio* for this subgroup of complex cases. The recent literature consists of small case series and expert recommendations (6–8). However, no current clinical trials or guideline recommendation exist to provide an evidence-based approach for those patients with a persisting reduction of quality of life.

This review aims to highlight the preoperative diagnostic steps and provides an overview of the current medical literature according to different surgical approaches and possibly solutions for patients requiring a urinary diversion as an *ultima ratio* due to their prolonged ordeal after prostate cancer treatment.

Literature review was performed separately by two authors of the study (BH, MW). Inclusion criteria were articles published between 1994 and 2021, using “urinary diversion,” “end stage bladder,” “devastated bladder outlet,” “complications prostate cancer” as search terms. Articles written in other language than English or German were excluded from further consideration.

Urinary diversion is defined as a surgically applied continent or incontinent mechanism for urine release after functional or disease-specific requirement of surgical intervention and removement of the natural anatomy of the urinary tract system. Foley catheterization and percutaneous nephrostomies are usually included in this definition (9). However, this review will mainly focus on long-lasting, definite types of urinary diversion.

Fundamental considerations regarding a continent vs. an incontinent-based urinary diversion have to be made in accordance with patient's age, comorbidities, manual dexterity, and cognitive ability (9). Table 1 summarizes the most common types of urinary diversions and their functional outcomes in terms of postoperative expected continence.

**Table 1:** Outline of the most commonly used urinary diversion types divided by the postoperative expected continence type.

**Urinary diversion**	
**Continent types**	**Non-continent types**
- Suprapubic vesicostomy (minimal -invasive) - Appendicovesicostomy - Ileovesicostomy - Cystoplasty with simultaneous Ileocecal bladder-augmentation - Ureterosigmoidostomy - Colon-pouch (Mainz-pouch I/III, Indiana pouch)	- Ureterosigmoidostomy - Cystectomy followed by ureterocutaneostomies - Cystectomy followed by Ileum/colon-conduit

Within continent urinary diversions, different surgical approaches are known concerning the type of bowel used and different types of continence-mechanisms, either based on a flap-valve principle (Mitrofanoff appendicovesicostomy, Yang-Monti-Channel) or nipple-valve principle (Intussuscepted ileal channel) (8, 10, 11). It is of note, that some findings are drawn from small case studies and derived partly from pediatric patients.

Prerequisites for quality of life are: a sufficient capacity (and if possible, well-contracted), reservoir (storage), a competent sphincteric mechanism and an unobstructed outlet (emptying). Especially radiation can damage all these three components without the tendency of healing over time. In this case urinary diversion remains as only solution. To avoid further complications of any surgical reconstructive procedure, one should take care not to use tissue which has been exposed to radiation, or use healthy vital tissue for interposition (e.g., greater omentum, pedunculated rectus or gracilis muscle flaps) (10, 12). Attention should be given to the bladder neck area. In any circumstances, surgical closure of the bladder neck should be performed in order to minimize risks of vesicourethral fistulae (13). Furthermore, following cases studies and experts opinions, tissue interposition should be performed to minimize complications such as vesicourethral fistulae (8, 9, 14). For example, the greater omentum or Musculus rectus abdominis/gracilis have been used as a vital tissue interposition with sufficient clinical results. Opposed to a bladder neck closure, a perineal closure of the distal urethra can be performed in a subgroup of patients, who are not eligible for a transabdominal approach and want to avoid an abdominal operation, especially after radiation therapy (6).

It is important to mention, that above mentioned general comments regarding surgical procedures must not be seen as a strict guideline. They should rather be considered as a pool of recommendations which can support decision making for both surgeons and patients. On behalf of the YAU Special edition “Sequelae of prostate Cancer Therapy: Avoidance Strategies and Management options,” a detailed summarization of different complications, which can lead to a urinary diversion at the far end of conservative treatment, will be discussed here.

## Osteomyelitis of the Symphysis Pubis/Osteitis Pubis Following Urosymphyseal Fistula

### Definition, Etiology, and Clinical Presentation

Osteomyelitis of the symphysis pubis and osteitis pubis are two rare complications following prostate cancer treatment (15). Since osteitis pubis is defined as a painful inflammatory process resulting in bone destruction of the symphysis margins, osteomyelitis of the symphysis is additionally associated with a detection of bacteria in bone cultures (16). In the vast majority of cases, urosymphyseal fistulae can be observed as the cause for this rare and debilitating diseases and complications after prostate cancer treatment (17).

Diagnosis of these progressive inflammation processes can be difficult and may prolong patients' suffering. Patients can also present with non-urological symptoms such as unspecific lower bowel/suprapubic pain, limitations in mobility, and generally reduced quality of life (18, 19). Chronic pubic pain is a common symptom following surgical and non-surgical prostate cancer treatment. However, prolonged episodes of pain should raise suspicion and physicians should consider the above-mentioned diseases as its origin for the patient's suffering. Furthermore, recurrent urinary tract infections and voiding discomfort can also occur as additional symptoms (17).

### Diagnosis and Investigations

**Figure 1:**
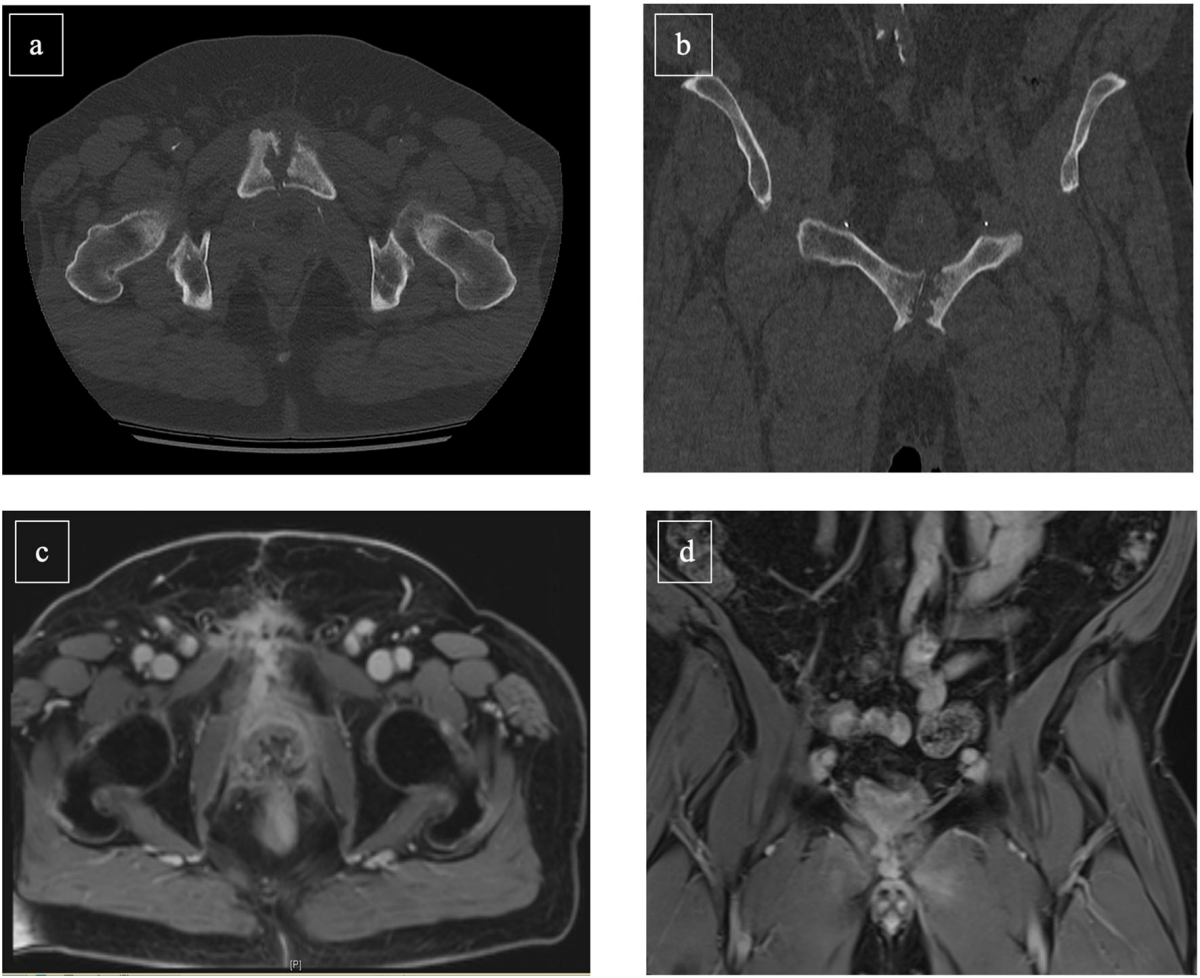
Abdominal CT scan [**(a)**: transversal, **(b)**: frontal] and MR-Imaging [**(c)**: transversal, **(d)**: frontal] of a 56-year-old prostate cancer patient suffering osteomyelitis following photon-beam radiation therapy of the prostate (2017) and salvage radical prostatectomy with persistent insufficient anastomosis (2019). Cystectomy with Mainz-I pouch with appendix-nipple was successful performed. Furthermore, symphyseal resection and omentus major flap was simultaneously achieved.

When osteomyelitis of the symphysis/pubis and urosymphyseal fistula is suspected, clinical assessments should include physical examination, ultrasound diagnostics, and blood testings. Additionally, urethrocystoscopy and urodynamics are important diagnostic tools to evaluate size and location of a fistula, its relationship to the orifices and a normal function of the urinary tract. Moreover, concomitant bladder neck contractures can be excluded by above-mentioned clinical assessments. Furthermore, bladder capacity and sphincteric competence should ideally be assessed within those diagnostic methods (14). Magnetic resonance imaging (MRI) with contrast agent provides currently the most accurate diagnostic modality for the confirmation or rejection of urosymphyseal fistula (20, 21). Conventional radiographs can be additionally performed, if involvement of bone structures cannot be sufficiently assessed by prior MRI (Figure 1). Moreover, it should be considered that a delay in diagnostics can cause a progress in inflammation and infection and evade into the perineum, scrotum, groin, or thigh resulting in abscesses and chronically discharging sinuses (14).

### Epidemiology

The current literature of body is scarce including only few sporadic case reports and small heterogenous studies about urosymphyseal fistula including 13–36 patients (14–16). It is noteworthy to mention, that the vast majority of patients suffering from osteomyelitis with concomitant urosymphyseal fistula had a history of definitive radiation therapy for initial prostate cancer treatment. In a case review by Kahokehr et al. (15) including 36 patients between 2012 and 2019 and addressing the prevalence of urosymphyseal fistula, solely three patients (8.3%) who underwent extirpative surgery for urosymphyseal fistula, were initially treated with a radical prostatectomy for primary prostate cancer. The vast majority of patients (91.7%) received either radiation therapy or combination of radiation therapy and radical prostatectomy (15). These findings are in an agreement with the results from a single-center case series by Bugeja et al. (14) (*n* = 16), where all urosymphyseal fistula patients (100%) were initially treated with radiation therapy for prostate cancer disease.

It is also of note that few cases of urosymphyseal fistula and concomitant osteomyelitis were observed in patients undergoing salvage focal therapy for prostate cancer treatment, such as HIFU, and palliative transurethral resection of the prostate following initial radiation therapy of prostate cancer treatment (19, 22, 23).

### Management

The vast majority of patients fail to respond to conservative management for urosymphyseal fistula, including analgesia, antibiotics, and intermittent urine diversion by a urethral or suprapubic catheter (14). After failure of conservative management, a subsequent radical surgical management (urinary diversion and/or debridement) with periinterventional antibiotic therapy is mostly applied. Nosé et al. (24) demonstrated in a case series of 33 patients who underwent extirpative surgery with urinary diversion for urosymphyseal fistula that urine culture correlated in 63% with bone culture results in patients. In consequence, the radical surgical approach normally includes the resection of the pubic symphysis joint and all affected pubic bone in combination with fistula excision and interposition of healthy tissue (14, 24).

Following a retrospective review (*n* = 36) published by Kahokehr et al., (15) 89% of patients suffering from urosymphyseal fistula following initial prostate cancer treatment, harbored osteomyelitis in histological analysis. The majority of these patients had a history of radiation therapy (92%). Here, all patients underwent extirpative debridement of the pubic bone. Noteworthy, concurrent cystectomy with urinary diversion was performed in 92% and two patients had already undergone cystectomy prior to presentation. Conversely, the bladder could be preserved solely in one patient. Interestingly, this patient did not have a history of radiation (15). In contrast, Bugeja et al. presented a case series of 16 patients being treated for urosymphyseal fistula, in which reconstruction by salvage prostatectomy and substitution/augmentation cystoplasty was successful in seven patients (47%). Conversely, cystectomy and ileum conduit were the preferred urinary diversions for eight patients (53%). Mundy et al. stated, that the ability to successfully reconstruct the lower urinary tract is strongly related to bladder capacity and compliance, which are commonly significantly reduced after pelvic radiation (25). Both case series emphasized the importance of pubic bone resection, tissue interpositioning and, if applicable, bladder neck closure at time of urosymphyseal fistula surgery. Table 2 outlines the most important characteristics which play a determining factor whether reconstructive surgery or cystectomy followed by a urinary diversion might represent the more suitable surgical approach. Moreover, it should be mentioned that in cases of bone involvement, interdisciplinary approaches including Urologists, Microbiologists, and Orthopedic surgeons should be targeted.

**Table 2:** Important patient and anatomical characteristics determining reconstructive surgery vs. cystectomy including urinary diversion in patients with devasted bladder outlets after prostate cancer treatment.

**Patient characteristics**	**Anatomical and functional characteristics**
- Prostate cancer treatment (radical prostatectomy, external beam radiation therapy, high intensity focused ultrasound, focal therapy [cryo/brachytherapy]) - Prostate cancer status (cancer-free, local/distant recurrence, progressive disease) - Age - Comorbidities - Body habitus, Body Mass Index - Performance status - Mental capacity/motivation	- Status of bladder (bladder capacity, compliance) - Size and location of fistula - Prostate organ still *in situ* - Presence of concomitant bladder neck contracture - Presence of pre-sacral cavity - Concomitant fistula into the rectum - Length of proximal bulbar urethra available to anastomose bladder/neobladder onto

## Radiation Therapy-Associated Bladder Toxicity

### Definition, Etiology, and Clinical Presentation

Radiation therapy of the pelvic structures is in general associated with bladder toxicity as a specific type of iatrogenic damage of the bladder. This holds especially true for prostate cancer treatment, which is usually performed with 74–80 Gy in primary prostate cancer treatment (2, 26). Besides urinary tract infections following radiation therapy, radiation-induced cystitis is a common challenging side effect of radiation therapy. This radiation therapy-induced cystitis is mainly related to DNA-damage associated endarteritis, including bladder hypoperfusion, which leads to mucosal atrophy, hypocellularity, and hypovascularity (27, 28). Patients suffering from hemorrhagic cystitis can present with mild intermittent hematuria. Conversely, also recurrent, progressive, and uncontrollable bleeding can end in life threatening situations.

### Diagnosis and Investigations

Radiation therapy-induced cystitis is a chronic condition characterized by urinary frequency, dysuria, incontinence, and pelvic pain. Hemorrhagic cystitis is a subtype, referred to when hematuria is present and is usually described as a late toxicity effect (29). Reduced bladder capacity and compliance and occurrence of secondary bladder malignancy can be also observed (30, 31). The existence of all of these symptoms occurring simultaneously (reduced bladder capacity, pain persistence and recurrent hematuria) are marked as a so called *end-stage bladder*, demonstrating the maximum expression of radiation-associated bladder toxicity (6). Diagnostic work-up should contain the exclusion of other symptoms-related side effects (29). Besides clinical examination and urine analyses, diagnostic urethrocystoscopy should be performed for visual assessment and rule-out intravesical malignancies. In doubt, urological imaging (computed tomography or magnetic resonance Imaging) can additionally be performed (32).

### Epidemiology

The reported incidence of radiation-induced cystitis varies from 23 to 80%, depending on the definition of cystitis, types, and dosage of radiation therapy and the studies observation period (32, 33). The median period for developing radiation-induced cystitis is given with 36 months after radiation therapy for prostate cancer treatment. Nonetheless, acute bladder toxicity symptoms can also occur in a shorter period of time (29, 34). Incidences of hemorrhagic cystitis range from 2.6 to 12.1% in prostate cancer patients primary treated with radiation therapy, depending on the duration of follow up (35–37). The median time to the appearance of hemorrhagic cystitis range from 48 to 79 months in the current literature (37, 38).

### Management

Treatment of radiation-associated bladder toxicity depends on the severity and derogation of quality of life for patients. It has to be emphasized, that sufficient randomized trials are lacking and most treatment options are based on small sample size (29). Suggested treatment options comprise simple bladder irrigation, cystoscopic fulguration, intravesical treatment with alum or formalin, hydrodistention, or hyperbaric oxygen therapy (39). Internal iliacal artery embolization can be taken into consideration if hematuria is intractable and contraindication exist regarding a definite surgical solution with cystectomy. However, success rate vary widely and a non-neglectable amount of patients is prone to further interventions (40).

Cystectomy with urinary diversion can be seen as the last resort of *end-stage bladder* following radiation therapy and reduced quality of life due to persisting patients' suffering. Urinary diversions in form of (ileum)-conduit and ureterocutaneostomies were preferred types of urinary diversion in most studies (41–43). In a retrospective review by Faris et al. (*n* = 30), analyzing treatment patterns of patients undergoing urinary diversion following radiation therapy for prostate cancer, four out of five *end-stage bladder* patients (80%) underwent cystectomy with conduit diversion. Conversely, suprapubic catheter was placed in the remaining 20%. Similar distributions could be observed for patients suffering *devastated-bladder outlet* or a combination of both in this case series (41). In line with these findings, Sack et al. demonstrated in a case series of 15 patients undergoing urinary diversion following radiation therapy of prostate cancer, that cysto(-prostat)ectomy followed by a ileum conduit was the most frequently administered type of urinary diversion in this cohort (88%) (43). Ureteroileal stricture is more often seen in irradiated patients undergoing ileal conduit as a form of urinary diversion. Gontero et al. (44) demonstrated an ureteroileal stricture rate of 9.4%, whereas, non-irradiated control groups presented with significant less rates (45). One should bear in mind, that this was a case series of 643 patients receiving a cystectomy with a radiation therapy due to different oncological tumors (prostate cancer, bladder cancer, colon cancer).

It is of note that technical developments of radiation therapy took place within the recent years with respect to more precise delivery of the dosage and hypofraction was introduced for the treatment of prostate cancer. These developments may hopefully translate into less occurrence of end-stage bladders in the future and makes it crucial for reassessment of the radiation therapy-related data in the following years.

## Urorectal and Vesicocutaneous Fistulae

### Definition, Etiology, and Clinical Presentation

Urorectal fistula is a well-known, but fortunately, uncommon complication of the treatment for prostate cancer with radical prostatectomy or radiation therapy (46). Besides radiation therapy (47) and iatrogenic damage of the rectum during radical prostatectomy (48), salvage prostatectomies after failure of radiation therapy (49), and a transperitoneal radical prostatectomy approach (47) are described risk factors to develop urorectal fistula. Especially, post-prostatectomy fistula often involve a direct track from vesicourethral anastomosis into the rectum (14). Radiation therapy increases the complexity of urorectal fistula, leaving the surrounding tissue ischemic and scared, often combined with cavitation. In general, radiation therapy-associated fistula tend to have a larger diameter and longer fistula-tracks (50). Common symptoms in regards to urorectal fistulae are pneumaturia (75%), faecaluria (63%), und recurrent urinary tract infections (57%) (51). Severe rectal or pelvic pain can furthermore be among the leading symptom (52).

### Diagnosis and Investigations

Diagnostic workup should include a thoroughly medical history taking and clinical examination. Furthermore, a retrograde urethrogram combined with a micturition-cystourography should be done. Standard, but mandatory, radiographical imaging must be performed in anterior-posterior and lateral recording in order to detect a potential fistula-track running dorsally (53). Additionally, diagnostic urethrocystoscopy seems essential to confirm and determine the size and location of the fistula and its relationship to the orifices and exclude concomitant urethral anomalies. To elucidate the length, size, and precise location of the fistula, rectoscopy, and contrast-agent based imaging of the rectum- and colon should also be performed (54). By using MRI, uncertainties, including potential concomitant fistula cavities and quality of surrounding tissue, can be ruled out prior to decision making for surgical treatment (50, 55).

### Epidemiology

The reported incidence of urorectal fistula is fairly uncommon and appears between 0.1 and 2% in recent literature (48, 56, 57). Patients undergoing salvage prostatectomy (58) or salvage HIFU-therapy (59) are at highest risk (1–3 and 5%, respectively) of developing an urorectal fistula. An extremely rare complication of fistula are vesicocutaneous fistulae following radiation therapy and reported solely in case reviews and are only included in this review for the sake of completeness (60, 61).

### Management

Spontaneous healing of urorectal fistulae following a conservative treatment is unlikely and should be critically discussed with the patient (62). Nevertheless, an intermittent suprapubic catheter should be inserted to minimize local irritation (63). Radiation-associated fistula tend to have even less chances of a spontaneous healing within a conservative management, due to the above mentioned pathophysiology (57).

Excision is the first step in the surgical treatment of urorectal fistulae and can be performed *via* different surgical approaches: Transanorectal sphincter splitting (York/Mason approach), peri-anal rectal advancement flap (Park approach), transabdominal, and perineal are established surgical procedures. Especially the two latter approaches reported sufficient success rates between 60 and 100% in case series, including 18 and 37 patients (55, 62, 64, 65). If possible, interposition of vital tissue (as above stated, e.g., Omentum flap, M. gracilis flap) should be performed and contribute to lower rates of fistula recurrences (66).

The effect of prior radiation therapy on the surgical outcome for urorectal fistulae was remarkably demonstrated by Linder et al. In their retrospective cases series of 42 patients diagnosed between 1998 and 2010, 16 patients with urorectal fistula had no history of radiation. Conversely, 26 patients were exposed to radiation therapy following prostate cancer treatment. Noteworthy, a primary repair (defined as surgical fistula excision and restoration of the natural urine outlet) was more frequently administered (94 vs. 21%) and more successful in the cohort of non-radiated patients (87 vs. 17%). Management of patients with prior radiation and urorectal fistula resulted very often in a permanent urinary diversion (93%) with concomitant permanent colostomy (86%) (52).

Irrespective the high success rates for successful primary repair of urorectal fistula in non-radiated patients, those with a history of radiation therapy are at high risk to fail a repair attempt and should be managed with a urinary diversion with or without a (temporal) bowel diversion (52). Furthermore, for urorectal fistula, a multidisciplinary approach is necessary for best treatment results and patient's care. Specifically, urologists, general surgeons, and dietary therapists should work hand in hand.

## Vesicourethral Anastomosis Stenosis, Bladder Neck Contracture, and Urethral Strictures

### Definition, Etiology, and Clinical Presentation

Vesicourethral anastomosis stenosis, bladder neck contracture (or also described as bladder neck stenosis) and urethral strictures can be seen as complications following all types of prostate cancer treatment (67). All complications can be seen as a result of luminal constriction caused by tissue fibrosis (5). The term “stricture” – according to recent definitions — is used if the narrowing part of the urethra is surrounded by corpus spongiosum, including fossa navicularis, penile, and bulbar urethra. All other locations with narrowed diameter are defined as “stenosis” (5). Unfortunately, the past literature has not been differentiating between vesicourethral anastomosis stenosis and bladder neck contracture precisely. It should be highlighted, that a differentiation between bladder neck contracture, which can occur after surgical procedures for benign prostatic hyperplasia and vesicourethral anastomosis stenosis after radical prostatectomy, is inevitable, since anatomy, recurrence rates and functional outcomes differ significantly (68, 69). Since a small subgroup of PCa patients might receive a palliative endoscopic procedure, bladder neck contracture and urethral strictures are listed as potential complications following PCa treatments, however, the majority of patients presenting with obstructive outlet following prostate cancer treatment will suffer of vesicourethral anastomosis stenosis. Patients suffering above mentioned post-prostate cancer treatment complications generally present with lower urinary tract symptoms, recurrent urinary tract infections, and slowing of the urinary stream in uroflowmetry (70). Furthermore, irritative symptoms with subjective residual urine are described (71).

### Diagnosis and Investigations

**Figure 2:**
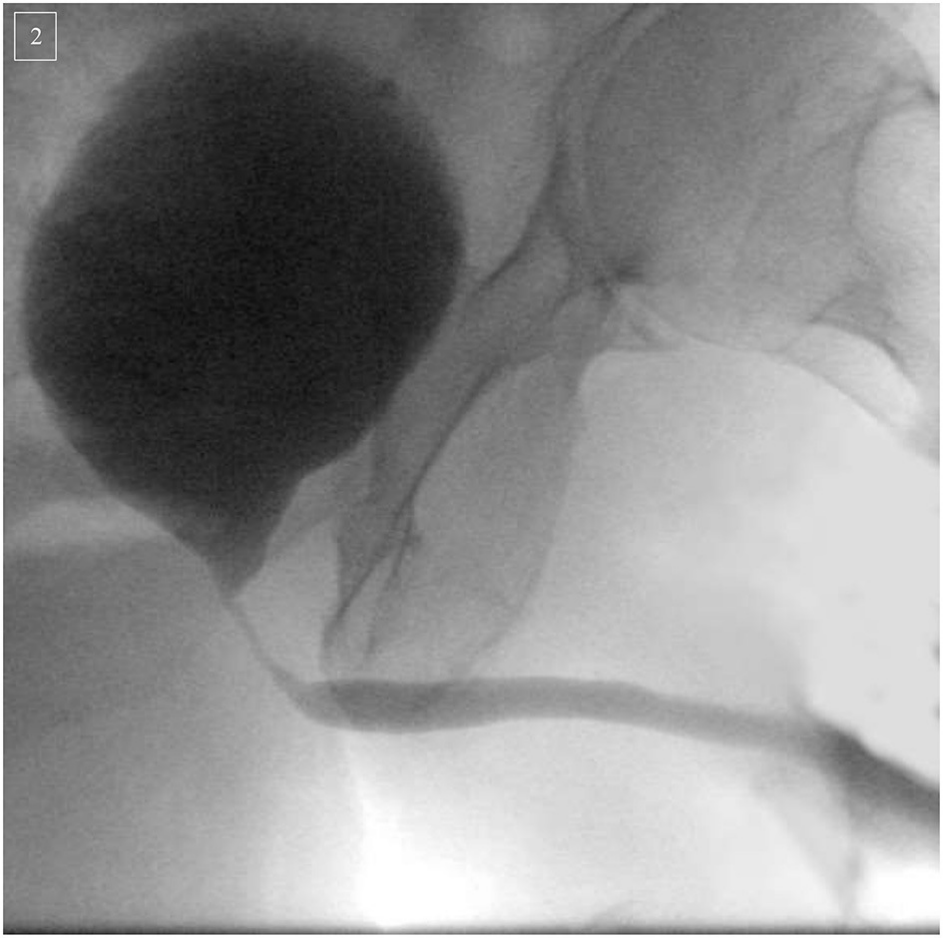
Micturition-cystourography of a 74-year-old patient suffering of an infra/intersphincteric urethral stricture following robotic-assisted radical prostatectomy (2016) and adjuvant radiation (2017) therapy for prostate cancer. Urethroplasty with mucosal ventral-onlay graft was successfully performed.

The diagnostic work up begins with a thorough history and physical examination. The history should elicit prior (endoscopic) treatments, history of radiation therapy and presence of urinary incontinence. Laboratory evaluation consists of urine analysis to rule out hematuria or urinary tract infection (72). Additionally, uroflowmetry, measurement of post-void residual and evaluation of concomitant (in)continence should ideally be performed (63). More invasive diagnostic measurements should include diagnostic urethrocystoscopy and retrograde urethrogram combined with a micturition-cystourography (Figure 2). In certain instances, urodynamic testing can give further insight into the bladder capacity/compliance (13).

### Epidemiology

Due to incongruent definitions and insufficient data, incidences for each localization can only vaguely be assessed. Based on the large-scale North American CaPSURE database, the overall incidence of urethral strictures and stenoses treatments following prostate cancer therapy, is 5.2% in the United States (73). However, no such large-scale databased analyses are currently available for European patients. In consequence, further, epidemiological research is needed to provide and improve information about the risk of the mentioned post-prostate cancer treatment related complications. The incidence of radiation therapy-induced urethral strictures and stenoses varies between 0 and 18% and is also affected by the delivered dosage and sort of radiation therapy (74). Specifically, in a review of more than 16,000 patients, the prevalence of strictures and stenoses was 2% after external beam radiation therapy (EBRT), 2% after brachytherapy and 5% after combination therapy with an median follow-up of 4 years (75). Other studies have reported an incidence rate of 12% urethral strictures or stenoses following a combined radiation therapy (EBRT plus brachytherapy) with an median follow up of 5 years (76). The main affected location of the male urethra seems to be the bulbomembranous urethra, followed by the bladder neck (77). Following a study by Msezane et al. (78), incidences of vesicourethral anastomosis stenosis after open radical prostatectomy and robot-assisted radical prostatectomy are given with 5.1 vs. 1.4%. Notably, initial incidence of stenosis occurred in ~30% cases at the beginning era of radical prostatectomy several decades ago. Improvement of surgical techniques in the recent years have been translated into lower stenosis rates in the recent decades. Surgical-induced stenoses occur mostly within 12 months after radical prostatectomy. Conversely, radiation therapy-induced strictures and stenoses tend to occur later on and in a more insidious fashion, up to 2–3 years after radiation therapy for prostate cancer treatment (73). Those specific time information have to be taken into account by physicians, when stricture/stenosis is suspected.

### Management

For the specific treatment of vesicourethral anastomosis stenosis after prostate cancer treatment, several different surgical approaches can be applied. Besides endoscopic dilatation, incision, or resection, open urethroplasty is a well-established surgical approach with satisfying clinical results and postoperative quality of life (79). It has to be mentioned, that results of urethroplasty in patients following radiation therapy tend to be less promising, but still remain the most favorable treatment option (71). Patients have to be informed prior to surgery, that by treating a stenosis a “hidden” incontinence can be demasked. Caused by the occurrence of the stenosis, patients can be classified as pseudo-continent after especially radical prostatectomy treatment of prostate cancer. In the first course of stenosis with endoscopic treatment, high rates of recurrences occur and increase with the number of redo endoscopic procedures. However, even the current gold standard of urethroplasties cannot always avoid recurrences. In combination with sphincteric damage, this state is often referred to as “devastated outlet” and is challenging for urologists, as well as patients (5).

Definite surgical solutions include bladder preservation with the closure of bladder neck and vesicostomy (continent vs. incontinent) with or without bladder augmentation. In a retrospective review by Faris et al. (41) evaluating 30 patients undergoing urinary diversion following radiation therapy for prostate cancer, *devastated outlet*, or a combination of *devastated outlet plus end-stage bladder* were the underlying cause for urinary diversion in almost the half of the cases (47%). Patients underwent 4 to 5 operative interventions aimed at salvage of lower urinary tract function, before receiving urinary diversion. The majority of patients (75%) suffering of devastated outlet received a cystectomy with conduit as a urinary diversion in this case series (41). In line with this single-center review, Bassett et al. confirmed in a multi-center case series of 100 patients undergoing urinary diversion following radiation therapy, vesicourethral anastomosis stenosis, and urethral strictures was in half of the patients (52%) the underlying cause of urinary diversion. A further differentiation regarding the exact location was not performed, however. Predominantly, patients underwent cystectomy (83%) with a conduit (84%) as urinary diversion. Noteworthy, Grade 3a or greater Clavien-Dindo complications occurred in 35% (*n* = 31) of these men, including four deaths (80). Complication rates for urinary diversion after irradiated prostate cancer patients are considerable, yet pros and cons must be carefully weighed up for each patient. Therefore older, multimorbid patients might benefit using suprapubic urinary diversion with a permanent suprapubic catheter (81, 82).

## Urinary Incontinence

### Definition, Etiology, and Clinical Presentation

Many patients prior to prostate cancer treatment decision making are concerned of post-treatment urinary incontinence. It is proven that urinary incontinence increases the risk of anxiety and depression and is associated with a lower healthcare related quality of life (83). Incontinence after prostate cancer treatment includes stress incontinence, urge incontinence and mixed incontinence (84). Especially, urinary incontinence after radical prostatectomy is mostly based on stress incontinence. However, patients can also simultaneously develop urge incontinence, which is related to a detrusor overactivity (85). Since surgical techniques improved in the recent years, stress urinary incontinence is less frequently observed after radical prostatectomy (86).

Following radiation therapy of the prostate for prostate cancer treatment, inflammatory changes can lead to a nociceptive response that may manifest as bladder detrusor overactivity, resulting in a urge incontinence (87). Definitions regarding incontinence following prostate cancer treatment, vary throughout the medical literature. Most commonly, continence is defined by no usage or usage of only one safety pad/24 h. Other definitions focus on the amount of urine loss, defining 2 g of urine loss/24 h or less as continent (88). Involuntary und uncontrollable leakage of urine is one of the bothersome symptoms of urinary incontinence. Furthermore, recurrent urinary tract infections and incontinence-associated dermatitis can additionally occur (83).

### Diagnosis and Investigations

Diagnostic work-up should include the medical history with focus on potential pretreatment incontinence and risk-factors. Specifically, a thorough physical examination and evaluation of the severity and type of incontinence needs to be done. Besides a precise mictionary diary, validated tools such as questionnaires (ICIQ-UI SF, M-ISI) and pad-tests should be performed (89–91). Due to its replicability, the 24-h pad-test is stated to be the most accurate pad-test to quantify urinary incontinence (92). Additional urine analyses can also rule out the prevalence of urinary infection. Moreover, diagnostic urethrocystoscopy should be performed to visually examine the bulbomembraneous urethra, external sphincter, and vesicourethral anastomosis. Although, its routine adoption is controversial discussed, urodynamic investigations can be used to determine the maximum bladder capacity and degree of bladder overactivity (93), giving important insights into the underlying type of urinary incontinence. Due to continued recovery to continence up to 12 months following radical prostatectomy, urodynamics investigations probably should be performed not earlier than 12 months after surgery unless other urgent circumstances exist (93).

### Epidemiology

Depending on stringency of definition, as well as the time point of its assessment, reported rates of stress incontinence after radical prostatectomy range widely from 2.9 and 87% (93). Recent data suggest an average long-term stress urinary incontinence rate after robot-assisted radical prostatectomy of 8–16% with above mentioned variability based on definition, surgical technique and skill level (88, 94). A study by Nam et al. (95) investigated, that ~5% of radical prostatectomy (open and laparoscopic approach) will require artificial urinary sphincter or male sling within 15 years after prostate cancer treatment. Additionally, overactive bladder symptoms can be present in up to 77% of patients following radical prostatectomy. However, during the first year after prostate cancer treatment, most of these symptoms resolve spontaneously (96).

Following a study by Pinkawa et al. (97), radiation therapy-induced urinary incontinence (defined as usage of pads) ranges between 8 and 15% after 5 years of follow up. Due to different radiation therapy modalities, radiation dosage and differing follow-up periods, issuing a precise statement regarding incidence rates of urinary incontinence following radiation therapy, is difficult (98).

### Management

**Figure 3:**
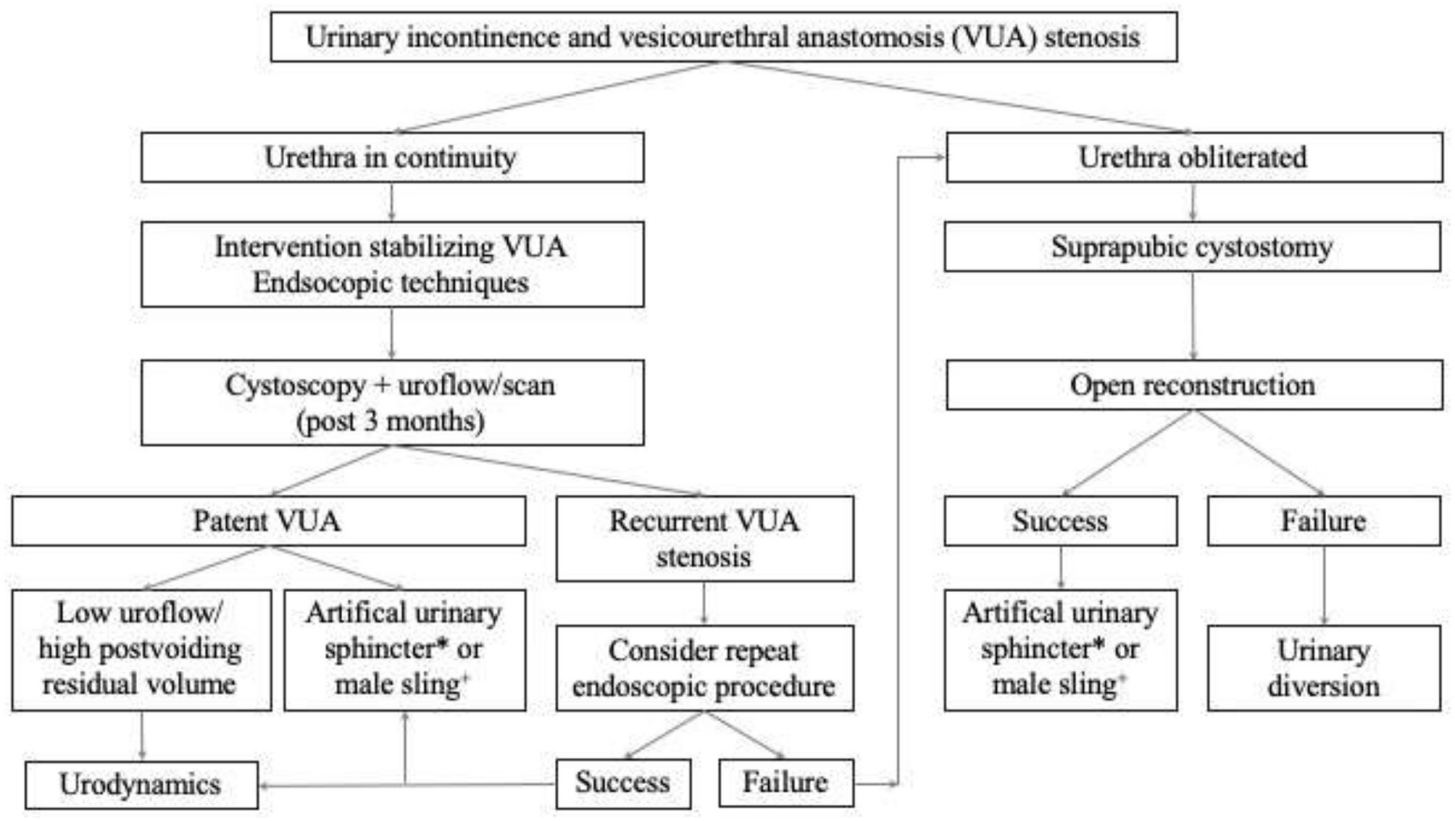
Suggested algorithm for the management of the incontinent patient with a concomitant vesicourethral anastomosis stenosis following prostate cancer treatment. *Artificial urinary sphincter preferably for patients with a history of radiation therapy. ^**+**^Male sling preferably for patients without a history of radiation therapy.

Management options of urinary incontinence have a wide range and can be stratified into conservative and surgical treatment options. If conservative management fails to sufficiently improve the incontinence situation and quality of life, subsequent surgical procedures need to be applied (84). Prior to surgery, concomitant problems, such as predominant overactive/small capacity bladder, vesicourethral anastomosis stenosis, or urethral strictures, must be excluded (84). Surgical treatment mainly includes the implantation of male sling systems or artificial urinary sphincter devices, the latter being the gold standard for males suffering of stress incontinence (99). Incontinent patients with a concomitant vesicourethral anastomosis stenosis should be managed gradually, treating the stenosis first. Figure 3 demonstrates a potential algorithm for this subgroup of patients. Success of an artificial urinary sphincter device is not only based on the expertise of the surgeon, but also on a precise and thorough selection of patients, who will be eligible and might benefit of it. Prior to sphincter implantation, concomitant vesicourethral anastomosis stenosis, and urethral stricture should be ruled out with a urethrocystoscopy which also helps to determine sphincteric damage (74). Furthermore, bladder detrusor overactivity must not be apparent during the first 300 ml of bladder filling in urodynamic investigations (99). Manual dexterity and mental ability for the usage of an artificial urinary sphincter must be ensured prior to device implantation (100). Due to clinical experience, a small, yet undeniable proportion of patients do not qualify for sphincteric implantation following above mentioned requirements. Some of them even present with a combination of urinary incontinence and vesicourethral anastomosis stenosis. Within this situation of a *devastated bladder outlet* urinary diversion can be seen as the final, yet definite treatment option. In different case series evaluating urinary diversions following prostate cancer treatment, *devastated bladder outlet* was among the major underlying causes to undergo urinary diversion. Cystectomy with ileum conduit was the preferred type of urinary diversion (80%) in a small case series of patients undergoing urinary diversion due to prostate cancer treatment complications (41). Cystectomy should usually be performed to prevent complications associated with leaving the bladder *in situ* with a closed bladder outlet (6).

## Radiation Therapy as a Risk Factor

Since a large body of evidence showed that pelvic surgery after radiation therapy is associated with a high risk of complications, we dedicated a specific paragraph on this important topic (44, 101–103). When it comes to the appropriate selection of tissue used for the urinary diversion, special caution needs to be administered in prostate cancer patient with an history of prior radiation therapy. In regards to the type of radiation therapy, collateral damage to the surrounding tissue is still often unavoidable and can cause progressive tissue ischemia, fibrosis and prolonged healing capabilities (104). From a urological point of view, usage of viable bowel outside the radiation field for urinary diversion, often referred as “stay away” principle, is elementary for a successful procedure (105). In line with published data, usage of non-irradiated intestine should be aimed at and preferably used in patients previously radiated in the pelvis, especially if a continent urinary diversion is seeked (41, 102, 106, 107).

Stolzenburg et al. (108) demonstrated in a case series of 24 female patients undergoing urinary diversion following radiation therapy, that usage of MAINZ-Pouch III can safely be performed with comparable outcomes to non-irradiated patients. As the MAINZ-Pouch III is in the upper abdomen, ureters can be cut at a very high level, thus ensuring an excellent blood supply. It has to be mentioned, that these patients were female patients mainly undergoing urinary diversion following a gynecological tumor treatment (108). By contrast, Wilkin et al. demonstrated in a long-time follow up of female patients with an INDIANA-Pouch following radiation therapy, feasibility of using both ileal and colon in irradiated patients. However, one has to highlight, that compared to non-irradiated patients, higher rates of complications and a significant increase in specific redo-surgery were observed (109). Above mentioned results can in general be transferred to prostate cancer patients undergoing urinary diversion strengthening the usage of non-irradiated tissue.

## Conclusion

With regards to an increasing global population, aging society, and improving prostate cancer treatment options, urologists will fortunately see more prostate cancer survivors than the generations before. New multimodal and focal therapies are likely to improve this positive and encouraging trend, but will also result in an increase of complications and side effects. Above painted scenarios of complications following prostate cancer treatment are statistically scarce, however, can be recalcitrant and frustrating for both patients and physicians. Decision-making should be performed in a multidisciplinary team and need to include the patient. Urinary diversion must be seen unarguably as a last resort. Even though, current literature lacks of reliable data regarding improvements in quality of life in form of PROMs, above mentioned case reports/study indicate beneficial improvements for patients' quality of life. Whenever possible, bowel for urinary diversion outside the field of prior radiation therapy should be used.

## Author Contributions

BH and MW: manuscript writing/editing, protocol/project development, and data analysis. SM: protocol/project development. LK: protocol/project development and manuscript writing/editing. All authors contributed to the article and approved the submitted version.

## Conflict of Interest

The authors declare that the research was conducted in the absence of any commercial or financial relationships that could be construed as a potential conflict of interest.

## Publisher's Note

All claims expressed in this article are solely those of the authors and do not necessarily represent those of their affiliated organizations, or those of the publisher, the editors and the reviewers. Any product that may be evaluated in this article, or claim that may be made by its manufacturer, is not guaranteed or endorsed by the publisher.
